# Neighborhood Disinvestment and Racial Disparities in Early Hypertension Onset Among Women

**DOI:** 10.1001/jamanetworkopen.2026.19845

**Published:** 2026-06-23

**Authors:** Elleni M. Hailu, Alexis N. Reeves, Tara McAlexander, Suzanne Judd, Michelle C. Odden

**Affiliations:** 1 Department of Pediatrics, School of Medicine, Stanford University, Palo Alto, California; 2 Institute of Health Policy Management and Evaluation, Dalla Lana School of Public Health, University of Toronto, Toronto, Ontario, Canada; 3 Women’s College Hospital, University of Toronto, Toronto, Ontario, Canada; 4 Department of Epidemiology and Biostatistics, Dornsife School of Public Health, Drexel University, Philadelphia, Pennsylvania; 5 Department of Biostatistics, School of Public Health, University of Alabama at Birmingham, Birmingham; 6 Department of Epidemiology and Population Health, School of Medicine, Stanford University, Palo Alto, California

## Abstract

**Importance:**

Although Black women are known to have higher hypertension rates than White women, longitudinal investigations of racial disparities in early hypertension onset and the role of neighborhood factors in shaping these differences are severely limited.

**Objective:**

To quantify racial disparities in early hypertension onset among women and examine the degree to which these disparities vary across levels of neighborhood socioeconomic disinvestment.

**Design, Setting, and Participants:**

This study used data from women participating in the Reasons for Geographic and Racial Differences in Stroke (REGARDS) study, a national longitudinal cohort study. Participants were recruited between 2003 and 2007. Data analyses were carried out from January to August 2025.

**Exposures:**

Self-reported race and ethnicity and level of neighborhood (ie, census-tract) socioeconomic disinvestment (low, moderate, or high), determined using a summary score of 6 socioeconomic indicators from US Census Bureau data.

**Main Outcomes and Measures:**

The primary outcome was age of hypertension onset, which was based on age at self-reported physician diagnosis or measured high blood pressure (≥140 mm/Hg systolic or ≥90 mm/Hg diastolic). Weibull accelerated failure time models with age as the time scale were used to estimate racial differences in age of hypertension onset, accounting for left, interval, and right censoring. Estimates were adjusted for individual-level sociodemographic characteristics and health-related factors. Models evaluating disparities across neighborhood disinvestment additionally accounted for neighborhood clustering and rurality.

**Results:**

There were 15 313 women included in the study (mean [SD] age at baseline, 64 [9.5] years), of which 7079 (46.2%) self-identified as Black and 8234 (53.8%) self-identified as White. Black women acquired hypertension a median of 9.6 (95% CI, 9.0 to 10.2) years earlier than White women, independent of sociodemographic factors. Inequities persisted across levels of neighborhood disinvestment. In neighborhoods with low disinvestment Black women had hypertension a median of 9.2 (8.2 to 10.3) years earlier than White women. Within highly disinvested neighborhoods, hypertension occurred a median of 8.0 (95% CI, 6.9 to 9.1) years earlier in Black than White women.

**Conclusions and Relevance:**

In this cohort study, earlier hypertension onset experienced by Black compared with White women persisted regardless of neighborhood context, even after controlling for a wide range of covariates.

## Key Points

**Question:**

How early does hypertension occur among Black compared with White women, and to what extent does neighborhood socioeconomic disinvestment modify disparities in early hypertension onset?

**Findings:**

In this cohort study of 15 313 women, Black women acquired hypertension a median of up to 9.6 years earlier than White women, with disparities persisting across levels of neighborhood disinvestment.

**Meaning:**

These findings suggest intervention strategies that address structural determinants earlier in the life course are needed to eliminate racial disparities in accelerated cardiovascular risk.

## Teaser

This cohort study evaluates racial disparities in early hypertension onset among women and examines the degree to which these disparities vary across levels of neighborhood socioeconomic disinvestment.

## Introduction

High blood pressure may be more strongly associated with cardiovascular disease mortality in women than in men; furthermore, trajectories of blood pressure increases have been found to be steeper for women than they are for men.^1,2^ Data also indicate that compared with men of similar age, older women have higher prevalence and lower control of hypertension.^3,4^ In addition to social stressors that uniquely burden women (eg, sexism, caregiving demands, and intimate-partner violence), female-specific cardiometabolic changes throughout the lifespan (eg, during pregnancy or menopausal transition) and gynecological disorders may exacerbate the risk and progression of hypertension-related morbidity.^5,6^ Yet, the unique pathophysiology of hypertension in women remains underinvestigated.

Moreover, there are substantial racial and ethnic inequities in hypertension among women. Black women experience higher hypertension prevalence and poorer hypertension control than White women, with differences persisting throughout the life span.^7,8,9,10^ Emerging evidence also indicates that Black women may acquire hypertension at earlier ages than their White counterparts.^11^ Early onset of hypertension has detrimental consequences for the development and progression of its related complications, likely driving increased cardiovascular disease burden among Black women compared with White women.^12,13,14^ In previous work, compared with hypertension-free individuals, those diagnosed with hypertension when aged less than 55 years had more than double the risk of cardiovascular disease mortality.^13,15^ However, existing research quantifying disparities in early hypertension onset is primarily limited to cross-sectional studies and does not consider sources of bias (eg, censoring) in using prevalent cases to characterize differences.

Furthermore, how structural determinants, such as neighborhood factors, contribute to inequities in early onset of cardiometabolic dysfunction is severely underexplored. Neighborhoods determine access to health-promoting amenities and psychosocial resources; and poor neighborhood conditions may drive physiological wear and tear and subsequently earlier disease onset.^16,17^ More specifically, racially and ethnically marginalized individuals are more likely to live in socioeconomically disinvested neighborhoods with limited cardiovascular health conducive resources.^18,19^ These environments may compound with other intersectional stressors (eg, gendered racism), and disproportionately accelerate cardiovascular risk in Black women.^16,20,21,22,23^ However, how neighborhood socioeconomic contexts shape Black-White inequities in early hypertension onset in women remains unknown, which is necessary to inform early prevention strategies.

To address these gaps, we quantified Black-White inequities in early hypertension onset among women and examined the degree to which neighborhood socioeconomic disinvestment modifies these inequities. We leveraged a large national longitudinal cohort study and applied analytic methods that account for left, right, and interval censoring. By appropriately accounting for censoring, our estimates are able to more accurately approximate inequities in early hypertension onset.^11^

## Methods

### Study Population

Data are from the Reasons for Geographic and Racial Differences in Stroke (REGARDS) study. A detailed description of the cohort is available elsewhere.^24^ In brief, REGARDS is a longitudinal study that recruited 30 183 non-Hispanic Black and White adults aged 45 years or older from the 48 contiguous US states and the District of Columbia between the years 2003 and 2007. Two in-home examinations have occurred: 1 at study initiation and another between 2013 and 2016. The current analyses are restricted to female REGARDS participants. Participants missing observations on study variables were excluded from this study. We also excluded participants reporting hypertension diagnosis before age 10 years, given that childhood hypertension may not be reliably diagnosed.^25^ See eFigure 1 in Supplement 1 for details regarding the final analytic sample. All participating institutional review boards approved this study, and participants provided written informed consent. This study adheres to the Strengthening the Reporting of Observational Studies in Epidemiology (STROBE) reporting guidelines.

### Race

Self-reported race was collected during the baseline REGARDS examination, where participants identified their race (non-Hispanic Black or non-Hispanic White). We examine race as a sociopolitical construct that indicates racial marginalization.^19^

### Neighborhood Socioeconomic Disinvestment

Neighborhood (census tract) level socioeconomic disinvestment at baseline was measured via a widely used neighborhood socioeconomic status score that has been shown to influence cardiovascular risk.^20,26^ This composite score is a sum of *z* scores calculated across 6 socioeconomic domains, including median household income; median value of housing units; percentage of households receiving interest, dividends, or net rental income; percentage of adults 25 years or older who had completed high school; percentage of adults 25 years or older who had a bachelor’s degree; and percentage of adults 16 years or older in executive, managerial, or professional occupations. We linked this score, created from the 2000 US Decennial Census data, to participants’ residential census tracts collected at baseline. We categorized participants into tertiles of this measure, which were reverse coded to indicate disinvestment levels (low, moderate, or high). We conceptualize neighborhood disinvestment as a structural process stemming from decisions and policies that pattern the uneven distribution of resources across neighborhoods, leading to concentrated disadvantage (ie, low neighborhood socioeconomic status).

### Study Outcome

Age of hypertension onset was evaluated based on participant self-report and age at measured high blood pressure during study observation. At both in-person examinations, participants were asked if they had been diagnosed with hypertension, and those with a physician diagnosis were prompted to report the age (in years) at which they received diagnosis. During the first examination, participants reported exact age or age intervals (10-19, 20-29, 30-39, 40-49, 50-59, 60-69, 70-79, 80-89, 90-99 years) of diagnosis, and during the second examination, age at diagnosis was reported based on the previously specified intervals. For participants who reported to not have been diagnosed with hypertension, we determined their first study encounter with measured systolic blood pressure of 140 mm/Hg or higher or diastolic blood pressure of 90 mm/Hg or higher.

### Covariates

We considered the following variables, selected based on prior research (eFigure 2 in Supplement 1) and collected at baseline: income (<$20 000, $20 000-34,999, $35 000-74,999, ≥$75 000, or unknown), education (less than high school, high school graduate, some college, or college graduate and above), employment status (employed, unemployed, retired, or unknown), exercise frequency per week (none, 1-3 times, or ≥4 times), health care coverage (no or yes), body mass index (BMI), pack years of cigarette smoking, Center for Epidemiologic Studies Depression (CES-D) score, and rurality (rural or urban) determined from the US Department of Agriculture’s Rural-Urban Commuting Area codes from the year 2000 and linked with residential census tracts at baseline.^27,28^

### Statistical Analysis

We first calculated descriptive statistics to determine distribution of population characteristics overall and by race and neighborhood disinvestment. We then used accelerated failure time models with age as the timescale to determine age of hypertension onset comparing Black with White women, overall and stratified by neighborhood disinvestment. After evaluating different distributions, the Weibull distribution was found to fit the data best. We accounted for left, interval, and right censoring, defining 4 censoring groups (types 1-4). For self-reported hypertension diagnosis (censoring type 1), if participants’ exact diagnosis age was unknown, we used age ranges provided by participants for interval censoring. Left censoring was used for participants who reported no hypertension diagnosis (or those with unknown diagnosis status) but presented at the first examination with a high blood pressure reading (censoring type 2). For left-censored cases, based on prior work, models specified that hypertension occurred between age 20 and age at examination entry.^11^ We also used interval censoring if participants were free of hypertension at baseline (based on both self-report and blood pressure measurements), reported to not have been diagnosed between the first and second examination, but presented to the second examination with high blood pressure reading (censoring type 3). In this case, intervals were specified as participants’ age at first and second examination. Participants who did not develop hypertension during follow-up and those who were lost to follow-up were right censored (censoring type 4; See eTable 1 in Supplement 1 for censoring types and age intervals). In sensitivity analysis, to address potential misclassification of hypertension in adolescence, we treated participants reporting hypertension diagnosis before age 20 as left-censored cases, with intervals specifying hypertension occurrence between age 20 years and age at examination entry.

We adjusted for covariates sequentially. Our initial models were unadjusted (model 1). Model 2 adjusted for income, education, and employment, and model 3 additionally included exercise frequency, health care coverage, BMI, pack-years of smoking, and CES-D scores. Models evaluating associations across neighborhood disinvestment additionally included robust variance estimators to account for neighborhood clustering and further adjusted for rurality (model 3). We then used these models to estimate median age of hypertension onset for Black and White women and differences in estimated median age of hypertension onset comparing Black with White women. The delta method was used to calculate standard error estimates.^29^ In supplementary tables, we report age of hypertension onset ratios (eTable 2 in Supplement 1). Additional sensitivity analysis compared associations among those who did and did not move neighborhoods between study enrollment and second in-person examination. Analyses were completed in R version 4.4.41 (R Project for Statistical Computing) from January to August 2025.

## Results

### Descriptive Results

Among the 15 313 women included in this study (representing 78 970 person-years among participants with complete follow-up; mean [SD] baseline age, 64 [9.5] years), 7079 (46.2%) were Black and 8234 (53.8%) were White (Table 1). The majority of Black women (3503 participants [49.5%]) resided in highly disinvested neighborhoods, whereas most White women (3980 participants [48.3%]) lived in less disinvested neighborhoods. Throughout the entire follow-up period, hypertension was observed in 10 974 women in the overall sample (71.7%) and was more frequently observed among Black (5853 participants [82.7%]) than White (5121 participants [62.2%]) women. It also occurred more frequently among residents of highly disinvested neighborhoods (4013 participants [80.4%]) than residents of low disinvestment neighborhoods (3230 participants [62.5%]) (Table 2).

**Table 1.** Distribution of Population Characteristics Overall and by Race at Study Baseline, in the Reasons for Geographic and Racial Differences in Stroke Study, 2003-2007

	Participants, No. (%)		
	Overall (N = 15 313)	Black (n = 7079 [46.2])	White (n = 8234 [53.8])
Hypertension^a^	10 974 (71.7)	5853 (82.7)	5121 (62.2)
Neighborhood disinvestment			
Low	5165 (33.7)	1185 (16.7)	3980 (48.3)
Moderate	5154 (33.7)	2391 (33.8)	2763 (33.6)
High	4994 (32.6)	3503 (49.5)	1491 (18.1)
Age, y, mean (SD)	64.2 (9.5)	63.7 (9.3)	64.6 (9.6)
<60	5256 (34.3)	2556 (36.1)	2700 (32.8)
60-69	5659 (37.0)	2655 (37.5)	3004 (36.5)
≥70	4398 (28.7)	1868 (26.4)	2530 (30.7)
Education			
Less than high school	1973 (12.9)	1362 (19.2)	611 (7.4)
High school graduate	4255 (27.8)	1961 (27.7)	2294 (27.9)
Some college	4330 (28.3)	1920 (27.1)	2410 (29.3)
≥College graduate	4755 (31.1)	1836 (25.9)	2919 (35.5)
Income, US $			
<20 000	3491 (22.8)	2174 (30.7)	1317 (16.0)
20 000-34 999	3849 (25.1)	1856 (26.2)	1993 (24.2)
35 000-74 999	3992 (26.1)	1643 (23.2)	2349 (28.5)
≥75,000	1805 (11.8)	466 (6.6)	1339 (16.3)
Unknown	2176 (14.2)	940 (13.3)	1236 (15.0)
Employment status			
Employed	3968 (25.9)	1670 (23.6)	2298 (27.9)
Unemployed	2321 (15.2)	1039 (14.7)	1282 (15.6)
Retired	4900 (32.0)	2122 (30.0)	2778 (33.7)
Unknown	4124 (26.9)	2248 (31.8)	1876 (22.8)
Health care coverage			
No	1142 (7.5)	726 (10.3)	416 (5.1)
Yes	14 171 (92.5)	2545 (36.0)	7818 (94.9)
Exercise (times per week)			
None	5982 (39.1)	2894 (40.9)	3088 (37.5)
1-3	5531 (36.1)	2545 (36.0)	2986 (36.3)
≥4	3800 (24.8)	1640 (23.2)	2160 (26.2)
BMI, mean (SD)^b^	29.9 (6.9)	31.9 (7.1)	28.2 (6.3)
<25	3902 (25.5)	1080 (15.3)	2822 (34.3)
25-29.9	4836 (31.6)	2099 (29.7)	2737 (33.2)
≥30	6575 (42.9)	3900 (55.1)	2675 (32.5)
Pack-y of smoking, mean (SD)	9.4 (18.5)	7.6 (15.5)	11.0 (20.6)
0	8444 (55.1)	3888 (54.9)	4556 (55.3)
<10	2833 (18.5)	1519 (21.5)	1314 (16.0)
10-20	1219 (8.0)	634 (9.0)	585 (7.1)
>20	2817 (18.4)	1038 (14.7)	1779 (21.6)
Depression (CES-D), mean (SD)	1.4 (2.2)	1.6 (2.4)	1.2 (2.1)
No (0)	8303 (54.2)	3570 (50.4)	4733 (57.5)
Yes (≥1)	7010 (45.8)	3509 (49.6)	3501 (42.5)
Neighborhood rurality			
Urban	11 923 (77.9)	6044 (85.4)	5879 (71.4)
Rural	3390 (22.1)	1035 (14.6)	2355 (28.6)

Abbreviations: BMI, body mass index; CES-D, Center for Epidemiologic Studies Depression Scale.

^a^ Hypertension occurrence throughout entire study period.

^b^ Calculated as weight in kilograms divided by height in meters squared.

**Table 2.** Population Characteristics Across Levels of Neighborhood Disinvestment at Study Baseline, in the Reasons for Geographic and Racial Differences in Stroke Study, 2003 to 2007

Characteristic	Neighborhood disinvestment		
	Low	Moderate	High
Race			
Black	1185 (22.9)	2391 (46.4)	3503 (70.1)
White	3980 (77.1)	2763 (53.6)	1491 (29.9)
Hypertension^a^	3230 (62.5)	3731 (72.4)	4013 (80.4)
Age, y, mean (SD)	64.2 (9.5)	64.0 (9.5)	64.4 (9.4)
<60	1783 (34.5)	1822 (35.4)	1651 (33.1)
60-69	1868 (36.2)	1896 (36.8)	1895 (37.9)
≥70	1514 (29.3)	1436 (27.9)	1448 (29.0)
Education			
Less than high school	203 (3.9)	630 (12.2)	1140 (22.8)
High school graduate	1081 (20.9)	1507 (29.2)	1667 (33.4)
Some college	1539 (29.8)	1562 (30.3)	1229 (24.6)
≥College graduate	2342 (45.3)	1455 (28.2)	958 (19.2)
Income, US $			
<20 000	512 (9.9)	1127 (21.9)	1852 (37.1)
20 000-34 999	1144 (22.1)	1371 (26.6)	1334 (26.7)
35 000-$74 999	1677 (32.5)	1451 (28.2)	864 (17.3)
≥75 000	1117 (21.6)	463 (9.0)	225 (4.5)
Unknown	715 (13.8)	742 (14.4)	719 (14.4)
Employment status			
Employed	1545 (29.9)	1342 (26.0)	1081 (21.6)
Unemployed	625 (12.1)	795 (15.4)	901 (18.0)
Retired	1704 (33.0)	1714 (33.3)	1482 (29.7)
Unknown	1291 (25.0)	1303 (25.3)	1530 (30.6)
Health care coverage			
No	205 (4.0)	377 (7.3)	560 (11.2)
Yes	4960 (96.0)	4777 (92.7)	4434 (88.8)
Exercise (times per week)			
None	1807 (35.0)	2110 (40.9)	2065 (41.3)
1-3	1958 (37.9)	1858 (36.0)	1715 (34.3)
≥4	1400 (27.1)	1186 (23.0)	1214 (24.3)
BMI, mean (SD)^b^	28.2 (6.2)	30.3 (7.0)	31.3 (7.2)
<25	1751 (33.9)	1204 (23.4)	947 (19.0)
25-29.9	1757 (34.0)	1608 (31.2)	1471 (29.5)
≥30	1657 (32.1)	2342 (45.4)	2576 (51.6)
Pack-y of smoking, mean (SD)	9.2 (17.7)	9.8 (19.5)	9.2 (18.1)
0	2816 (54.5)	2853 (55.4)	2775 (55.6)
<10	977 (18.9)	915 (17.8)	941 (18.8)
10-20	402 (7.8)	427 (8.3)	390 (7.8)
>20	970 (18.8)	959 (18.6)	888 (17.8)
Depression (CES-D), mean (SD)	1.04 (1.9)	1.4 (2.3)	1.7 (2.5)
No (0)	3150 (61.0)	2747 (53.3)	2406 (48.2)
Yes (≥1)	2015 (39.0)	2407 (46.7)	2588 (51.8)
Neighborhood rurality			
Urban	4658 (90.2)	3832 (74.4)	3433 (68.7)
Rural	507 (9.8)	1322 (25.6)	1561 (31.3)

Abbreviations: BMI, body mass index; CES-D, Center for Epidemiologic Studies Depression Scale.

^a^ Hypertension occurrence throughout entire study period.

^b^ Calculated as weight in kilograms divided by height in meters squared.

### Regression Results

In the overall unadjusted model, Black women acquired hypertension significantly earlier than White women (difference in estimated median age of onset, −9.4; 95% CI, −9.9 to −8.8 years). Associations persisted in our model adjusting for education, income, and employment (difference, −9.6; 95% CI, −10.2 to −9.0 years) (Table 3). Estimated median age of hypertension onset in this model was 55.9 (95% CI, 55.1 to 56.7) years among Black women and 65.5 (95% CI, 64.6 to 66.4) years among White women (Table 3). Additionally controlling for health-related factors (eg, exercise) did not meaningfully change results for Black women (estimated median age, 55.8; 95% CI, 55.0 to 56.6 years). However, adjustment for these covariates decreased the estimate for White women (estimated median age, 62.8; 95% CI, 61.9 to 63.7 years), slightly attenuating differences (difference, −7.0; 95% CI, −7.6 to −6.4) (Table 3).

**Table 3.** Estimated Median Age of Hypertension Onset and Differences in Black and White Women Overall and Across Levels of Neighborhood Disinvestment, the Reasons for Geographic and Racial Differences in Stroke Study^a^

	Participants, No.	Model 1^b^		Model 2^c^		Model 3^d^	
		Age of onset, median (95% CI), y	Difference (95% CI), y	Age of onset, median (95% CI), y	Difference (95% CI), y	Age of onset, median (95% CI), y	Difference (95% CI), y
Overall							
Black	7079	53.7 (53.4 to 54.1)	−9.4 (−9.9 to −8.8)	55.9 (55.1 to 56.7)	−9.6 (−10.2 to −9.0)	55.8 (55.0 to 56.6)	−7.0 (−7.6 to −6.4)
White	8234	63.1 (62.7 to 63.5)	[Reference]	65.5 (64.6 to 66.4)	[Reference]	62.8 (61.9 to 63.7)	[Reference]
Neighborhood disinvestment^e^							
Low							
Black	1185	55.6 (54.8 to 56.5)	−9.4 (−10.4 to −8.3)	56.8 (55.6 to 58.1)	−9.2 (−10.3 to −8.2)	56.3 (54.9 to 57.6)	−6.6 (−7.7 to −5.6)
White	3980	65.0 (64.4 to 65.6)	[Reference]	66.1 (64.8 to 67.3)	[Reference]	62.9 (61.5 to 64.3)	[Reference]
Moderate							
Black	2391	53.9 (53.2 to 54.6)	−8.5 (−9.4 to −7.5)	56.3 (55.01 to 57.6)	−9.0 (−10.0 to −8.00)	56.6 (55.2 to 57.9)	−7.1 (−8.1 to −6.1)
White	2763	62.4 (61.7 to 63.1)	[Reference]	65.3 (63.8 to 66.8)	[Reference]	63.7 (62.1 to 65.3)	[Reference]
High							
Black	3503	52.6 (52.0 to 53.1)	−7.3 (−8.4 to −6.3)	54.4 (53.0 to 55.9)	−8.0 (−9.1 to −6.9)	55.0 (53.4 to 56.5)	−6.4 (−7.6 to −5.3)
White	1491	59.8 (59.0 to 60.8)	[Reference]	62.5 (60.7 to 64.2)	[Reference]	61.4 (59.5 to 63.3)	[Reference]

^a^ Estimates are from accelerated failure time models with age as the time scale; 95% CIs calculated using the delta method.

^b^ Model 1 is unadjusted.

^c^ Model 2 adjusts for income, education, and employment; median age estimations with income, education, and employment fixed at the sample majority ($35 000-$74 999, college graduate or more, and retired, respectively).

^d^ Model 3 further adjusts for exercise and health care coverage, BMI, pack-years of smoking, Center for Epidemiologic Studies Depression Scale (CES-D), median age estimations with exercise and health care coverage fixed at the sample majority (none and presence of health care coverage, respectively), sample mean for BMI (29.9; calculated as weight in kilograms divided by height in meters squared), pack-years of smoking (9.4), and CES-D (1.4), in addition to specifications listed for model 2.

^e^ Models additionally include robust variance estimators to account for clustering by census tract and fully adjusted models (model 3) additionally control for neighborhood rurality (estimations fixed at the sample majority—urban residence, in addition to specifications listed for overall models 2 and 3).

Racial disparities in early hypertension onset persisted across neighborhood disinvestment levels. Estimated median age of hypertension onset was 55.6 (95% CI, 54.8 to 56.5) years for Black women and 65.0 (95% CI, 64.4 to 65.6) years for White women in low disinvestment neighborhoods. In other words, Black women residing in less disinvested neighborhoods acquired hypertension a median of 9.4 (95% CI, 8.3 to 10.4) years earlier than White women in similar neighborhoods (Table 3). Results showed slight decreases in gaps with increasing disinvestment, with both Black and White women in highly disinvested neighborhoods experiencing earlier hypertension onset than their counterparts in less disinvested neighborhoods. In neighborhoods with high disinvestment, estimated median age of hypertension onset among Black women was 52.6 (95% CI, 52.0 to 53.1) years compared with 59.8 (95% CI, 59.0 to 60.8) years among White women, suggesting that in such neighborhoods, Black women acquired hypertension a median of 7.3 (95% CI, 6.3 to 8.4) years earlier than White women (Table 3). In models adjusting for sociodemographic characteristics, compared with White women in similar neighborhoods, Black women acquired hypertension a median of 9.2 (95% CI, 8.2 to 10.3) years earlier within low disinvestment neighborhoods and a median of 8.0 (95% CI, 6.9 to 9.1) years earlier in highly disinvested neighborhoods (Table 3). Further adjustment for health-related factors and rurality decreased racial differences by primarily attenuating estimated age of onset for White women. In neighborhoods with low disinvestment, Black women had hypertension a median of 6.6 (95% CI, 5.6 to 7.7) years earlier than White women (Table 3). Within neighborhoods experiencing high disinvestment, the difference in median age of hypertension onset was 6.4 (95% CI, 5.3 to 7.6) years earlier in Black than White women.

Results of our sensitivity analysis treating those reporting hypertension diagnosis before age 20 years as left-censored cases revealed similar results as our main analysis (eTable 3 in Supplement 1). Estimates among movers and nonmovers also remained generally similar to our main results (eTable 4 in Supplement 1).

## Discussion

This study used data from a large national cohort to examine racial disparities in early hypertension onset among women and the degree to which disparities vary across neighborhood socioeconomic disinvestment (ie, disadvantage). In models accounting for censoring and individual-level socioeconomic characteristics, Black women acquired hypertension a median of up to 9.6 years earlier than White women. Adjustment for health-related factors did not significantly alter differences. Racial disparities in early hypertension onset persisted across levels of neighborhood disinvestment, with gaps slightly decreasing with higher disinvestment. Findings highlight the need to address chronic racialized stressors earlier in the life course to eliminate racial inequities in accelerated cardiovascular risk.

Our results are corroborated by growing research showing that in contrast to White women, Black women may experience faster cellular aging and exhibit poorer subclinical cardiovascular risk profiles than White women.^30,31^ They are also consistent with recent findings documenting that stark Black-White disparities in cardiometabolic risk may begin as early as 45 years among women.^10^ Yet, existing work on accelerated health declines rarely examines disparities and unique racialized disease processes in women. Additionally, existing evidence on disparities in onset of chronic illnesses, such as hypertension, is primarily based on cross-sectional studies, warranting the need for studies that accurately examine timing of disease occurrence.^11,32^ As such, by evaluating early hypertension onset using longitudinal data and analytic methods accounting for censoring, our study contributes to the nascent empirical literature on accelerated physiologic decline among Black women. Results also provide additional context around persistent inequities in cardiovascular disease, which remains a major contributor to reduced longevity among Black individuals.^33^

In our study, the persistence of earlier hypertension onset among Black than White women, even after adjustment for individual-level characteristics, points to chronic stress pathways and accumulation of disadvantage throughout the life course as potential reasons behind accelerated physiological deterioration. Allostatic load frameworks and the weathering hypothesis further support the physiological salience of these mechanisms for the associations we observed.^23,34^ Compared with White women, Black women are likely to experience multiple forms of adversity across their lifespan at the nexus of both racism and sexism with far-reaching implications for their well-being.^35^ This includes heightened exposure to adverse early life environments, experiences of unfair treatment, and limited access to disease-mitigating resources. Studies also highlight that in response to such adversity, Black women often use high-effort coping strategies, which further exacerbate the biological toll from compounding stressors.^36^ This underscores the need for future studies that explicate unique psychosocial processes that may drive faster physiological wear and tear among Black women in relation to cardiometabolic disorders.^36^

We found that compared with residents of low disinvestment neighborhoods, both Black and White women in neighborhoods with high disinvestment generally experienced earlier hypertension onset. The influential impact of neighborhood conditions for cardiovascular risk has been well established.^20,21,22^ By determining the availability of health promoting amenities and infrastructural attributes (eg, walkability), residential environments drive residents’ engagement in healthy activities that prevent cardiovascular disease risk.^37^ This also extends to the accessibility of quality health care for early detection and treatment of risk factors, housing conditions, and environmental exposures.^38,39,40^ Neighborhoods also determine psychosocial contexts surrounding individuals, with physiologic implications for cardiovascular functioning.^41^ Our results documenting earlier hypertension onset in neighborhoods with reduced resources is aligned with these pathways and highlights the imperative for transforming residential environments to minimize hypertension risk.

Even though Black women had persistently earlier hypertension onset than White women regardless of neighborhood disinvestment, racial differences were slightly wider in low vs high disinvestment neighborhoods. One reason for this finding may be that most Black women in this study lived in disinvested neighborhoods (49%), whereas most White women resided in advantaged neighborhoods (48%). This indicates that Black women in our sample may be more likely to be exposed to more detrimental residential environments than their White counterparts, underscoring discriminatory policies and practices that have intertwined racial and economic segregation in the US.^42^ Furthermore, the protective benefits of neighborhood environments are known to translate differentially across populations.^43,44^ Prior studies have documented counterintuitive associations between neighborhood conditions and health among Black individuals, while similar neighborhoods have been found to be strongly linked to White individuals’ well-being.^45,46^ This is likely because even in neighborhoods that are less disadvantaged, Black individuals may experience discrimination and endure various forms of social stressors in and outside of their neighborhoods.^43^ This is supported by prevailing theories outlining widening Black-White health gaps with increasing socioeconomic status due to racism-related stress and the cost of social mobility for Black individuals, who face exceeding demands even in contexts conceptualized to be advantageous.^47,48,49^ Our findings highlight the need to incorporate intersectional perspectives to further unmask such heterogeneities.

### Limitations

Our results have several limitations. First, age of hypertension onset was partially determined based on self-reported diagnosis age. This may not accurately indicate hypertension onset and could instead more closely approximate time of hypertension status awareness, which may vary by race and ethnicity.^50,51^ Although we controlled for health care coverage, frequency of physician visits and other health care–seeking behaviors may have influenced our results. We also cannot rule out potential unmeasured confounding from several other factors. However, to best estimate racial inequities, we used analytic methods that appropriately account for different forms of censoring, especially left censoring, which more likely affects Black women.^11^ Future work using longitudinal studies of younger age groups is critical. Additionally, the measure of neighborhood socioeconomic status we used may not fully represent mechanisms of disinvestment in residential contexts. Future studies that comprehensively explicate processes related to place-based disinvestment are needed.^52^ Furthermore, in our study, neighborhood disinvestment and covariates were evaluated based on information collected at study enrollment and were not prospectively examined. These variables may be preceded by hypertension onset. A closer investigation of neighborhood trajectories and changes in individual-level characteristics across the life course is required to sufficiently capture underlying relationships. Relatedly, research examining how racism and other psychosocial stressors unique to Black women shape the differences we observed is warranted.

## Conclusions

To our knowledge, this study is the first of its kind to leverage well-characterized cohort data and methods accounting for various forms of censoring to document that Black women may acquire hypertension a median of approximately 9.6 years earlier than White women. Racial disparities persisted across levels of neighborhood disinvestment. Future research should disentangle neighborhood contexts throughout the life course and racialized chronic stressors to inform early interventions that promote cardiovascular health equity.

## Notes

Supplement 1. eFigure 1. Study Sample Flow Chart eFigure 2. Relationship Between Study Variables eTable 1. Range of Age Intervals Specified for Accelerated Failure Time Models to Account for Censoring, the Reasons for Geographic and Racial Differences in Stroke Study eTable 2. Age of Hypertension Onset Ratios Comparing Black to White Women, The Reasons for Geographic and Racial Differences in Stroke Study eTable 3. Estimated Median Age of Hypertension Onset and Differences in Black and White Women Overall and Across Levels of Neighborhood Disinvestment, the Reasons for Geographic and Racial Differences in Stroke Study eTable 4. Estimated Median Age of Hypertension Onset and Differences in Black and White Women Across Levels of Neighborhood Disinvestment by Moving Status, the Reasons for Geographic and Racial Differences in Stroke Study

Supplement 2. Data Sharing Statement
