# Supplementary material for: Neighborhood Disinvestment and Racial Disparities in Early Hypertension Onset Among Women
Source: JAMA Netw Open. 2026 Jun 23;9(6):e2619845. doi: 10.1001/jamanetworkopen.2026.19845 (PMC13291848; doi:10.1001/jamanetworkopen.2026.19845)
Supplement: Supplement 1. — eFigure 1. Study Sample Flow Chart eFigure 2. Relationship Between Study Variables eTable 1. Range of Age Intervals Specified for Accelerated Failure Time Models to Account for Censoring, the Reasons for Geographic and Racial Differences in Stroke Study eTable 2. Age of Hypertension Onset Ratios Comparing Black to White Women, The Reasons for Geographic and Racial Differences in Stroke Study eTable 3. Estimated Median Age of Hypertension Onset and Differences in Black and White Women Overall and Across Levels of Neighborhood Disinvestment, the Reasons for Geographic and Racial Differences in Stroke Study eTable 4. Estimated Median Age of Hypertension Onset and Differences in Black and White Women Across Levels of Neighborhood Disinvestment by Moving Status, the Reasons for Geographic and Racial Differences in Stroke Study [file jamanetwopen-e2619845-s001.pdf]

Supplemental Online Content

Hailu EM, Reeves AN, McAlexander T, Judd SE, Odden MC. Neighborhood disinvestment and racial disparities in early hypertension onset among women. *JAMA Netw Open*. 2026;9(6):e2619845. doi:10.1001/jamanetworkopen.2026.19845

eFigure 1. Study Sample Flow Chart

eFigure 2. Relationship Between Study Variables

eTable 1. Range of Age Intervals Specified for Accelerated Failure Time Models to Account for Censoring, the Reasons for Geographic and Racial Differences in Stroke Study

eTable 2. Age of Hypertension Onset Ratios Comparing Black to White Women, The Reasons for Geographic and Racial Differences in Stroke Study

eTable 3. Estimated Median Age of Hypertension Onset and Differences in Black and White Women Overall and Across Levels of Neighborhood Disinvestment, the Reasons for Geographic and Racial Differences in Stroke Study

eTable 4. Estimated Median Age of Hypertension Onset and Differences in Black and White Women Across Levels of Neighborhood Disinvestment by Moving Status, the Reasons for Geographic and Racial Differences in Stroke Study

This supplemental material has been provided by the authors to give readers additional information about their work.

eFigure 1. Study Sample Flow Chart

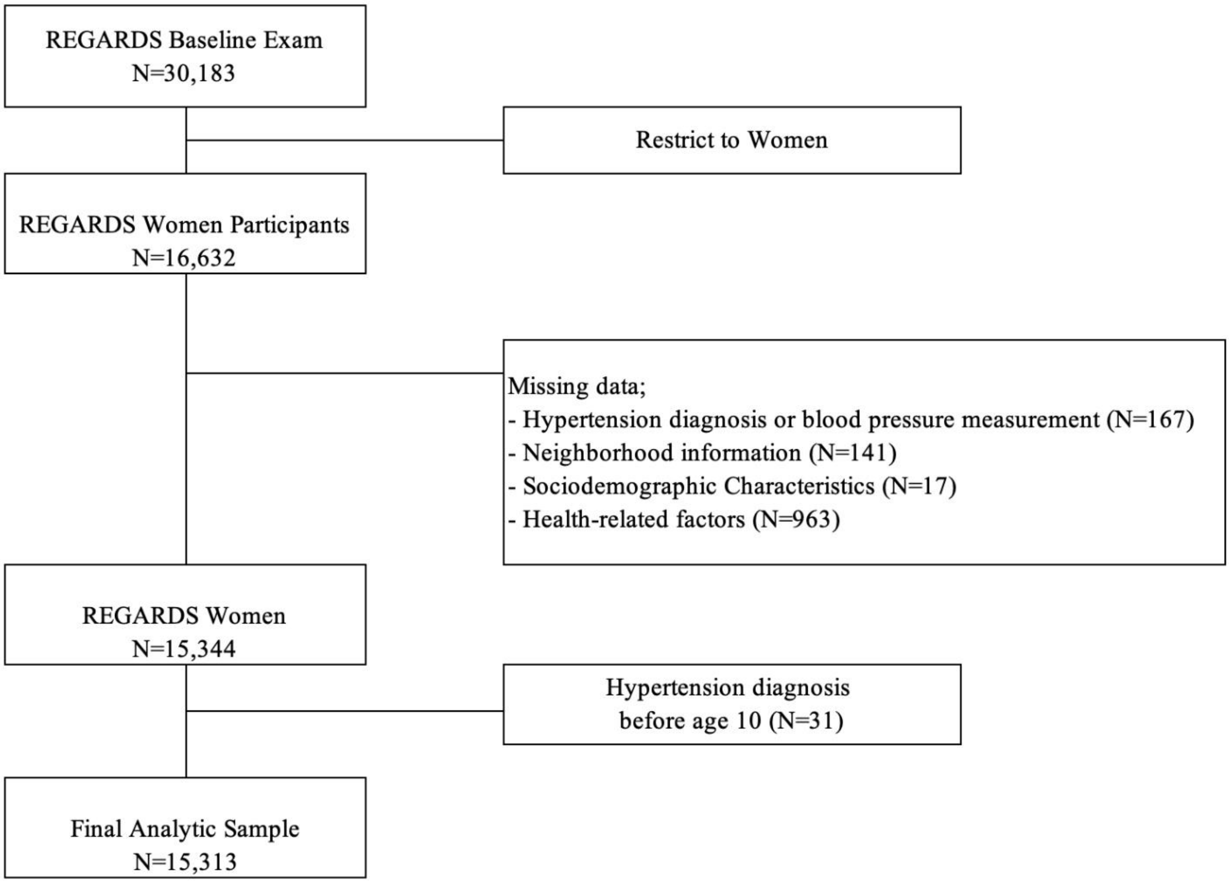

eFigure 2. Relationship Between Study Variables

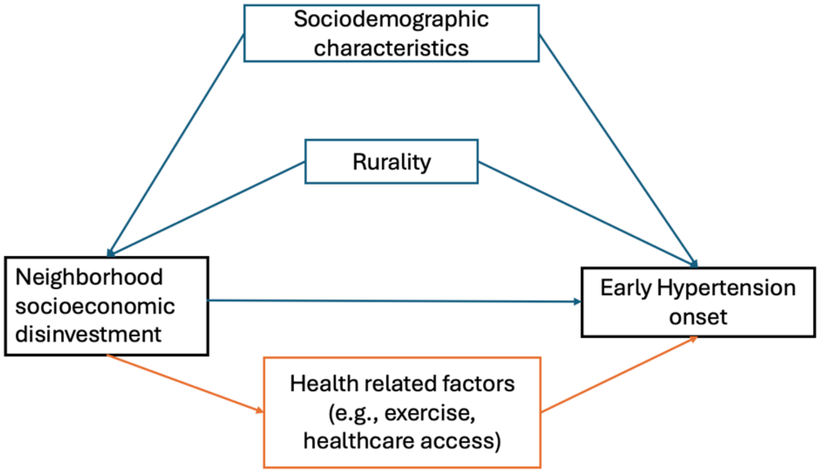

eTable 1. Range of Age Intervals Specified for Accelerated Failure Time Models to Account for Censoring, the Reasons for Geographic and Racial Differences in Stroke Study

	N	Interval Start Age			Interval End Age		
**Censoring Types**		**Min**	**Mean**	**Max**	**Min**	**Mean**	**Max**
Type 1: Self-reported diagnosis at either exam 1 or 2 [a]	10,181	10.0	48.1	93.0	10.0	54.9	99.0
Type 2: left censored (i.e. measured high blood pressure at exam 1)	656	20.0	20.0	20.0	45.0	66.1	91.0
Type 3: measured high blood pressure at exam 2	137	45.0	61.9	86.0	53.0	71.2	95.0
Type 4: right censored [b]	4,339	45.0	61.8	96.0	Inf	Inf	Inf

[a] Intervals for individuals reporting diagnosis were based on first recorded age (at either exam 1 or 2)

[b] Includes both who remained in the study and did not develop hypertension throughout follow-up (14.6% of total sample) and those who were lost to follow-up (13.8% of the total sample).

eTable 2. Age of Hypertension Onset Ratios Comparing Black to White Women, The Reasons for Geographic and Racial Differences in Stroke Study[a]

	N	Model 1[b]	Model 2[c]	Model 3[d]
		Age Ratio (95% CI)	Age Ratio (95% CI)	Age Ratio (95% CI)
**Overall**	15,313			
Black	7,079	0.85 (0.84,0.86)	0.85 (0.85,0.86)	0.89 (0.88,0.90)
White	8,234	Ref	Ref	Ref
**Neighborhood Disinvestment** [e]				
Low				
Black	1,185	0.86 (0.84,0.87)	0.86 (0.85,0.88)	0.89 (0.88,0.91)
White	3,980	Ref	Ref	Ref
Moderate				
Black	2,391	0.86 (0.85,0.88)	0.86 (0.85,0.88)	0.89 (0.87,0.90)
White	2,763	Ref	Ref	Ref
High				
Black	3,503	0.88 (0.86,0.89)	0.87 (0.86,0.89)	0.90 (0.88,0.91)
White	1,491	Ref	Ref	Ref

[a] Estimates are from accelerated failure time models with age as the time scale.
[b] Model 1 is unadjusted.
[c] Model 2 adjusts for income, education, and employment.
[d] Model 3 further adjusts for exercise, healthcare coverage, BMI, packyears of smoking, and CESD.
[e] Models additionally include robust variance estimators to account for clustering by census tract and fully adjusted models (Model 3) additionally control for neighborhood rurality.

eTable 3. Estimated Median Age of Hypertension Onset[a] and Differences in Black and White Women Overall and Across Levels of Neighborhood Disinvestment, the Reasons for Geographic and Racial Differences in Stroke Study[b]

	Model 1[c]		Model 2[d]		Model 3[e]	
	Age of Onset, Median (95% CI)	Difference (95% CI)	Age of onset, Median (95% CI)	Difference (95% CI)	Age of onset, Median (95% CI)	Difference (95% CI)
**Overall**						
Black	54.2 (53.9, 54.6)	-9.1 (-9.6, -8.5)	56.3 (55.6, 57.0)	-9.3 (-9.9, 8.8)	56.3 (55.5, 57.0)	-6.8 (-7.3, -6.2)
White	63.3 (62.9, 63.7)	Ref	65.6 (64.8, 66.5)	Ref	63.0 (62.2, 63.9)	Ref
**Neighborhood Disinvestment** [f]						
Low						
Black	55.8 (55.0, 56.7)	-9.3 (-10.3, -8.2)	57.1 (55.8, 58.3)	-9.1 (-10.1, -8.1)	56.5 (55.2, 57.8)	-6.6 (-7.6, -5.6)
White	65.1 (64.5, 65.7)	Ref	66.2 (64.9, 67.4)	Ref	63.1 (61.7, 64.4)	Ref
Moderate						
Black	54.4 (53.7, 55.1)	-8.2 (-9.1, -7.2)	56.7 (55.4, 57.9)	-8.7 (-9.6, -7.7)	56.9 (55.6, 58.3)	-6.9 (-7.8, -5.9)
White	62.6 (61.9, 63.3)	Ref	65.3 (63.9, 66.8)	Ref	63.8 (62.3, 65.4)	Ref
High						
Black	53.2 (52.7, 53.8)	-7.0 (-8.0, -6.0)	55.0 (53.6, 56.4)	-7.7 (-8.8, -6.6)	55.6 (54.1, 57.1)	-6.2 (-7.3, -5.1)
White	60.2 (59.3, 61.1)	Ref	62.7 (61.0, 64.4)	Ref	61.8 (59.9, 63.6)	Ref

[a] Participants reporting hypertension diagnosis before age 20 treated as left censored cases, with intervals specifying hypertension occurrence between age 20 and age at exam entry.

[b] Estimates are from accelerated failure time models with age as the time scale; 95% confidence intervals were calculated using the Delta Method.

[c] Model 1 is unadjusted.

[d] Model 2 adjusts for income, education, and employment status; median age estimations with income, education, and employment levels fixed at the sample majority ($35,000-74,999, college graduate or more, and retired, respectively).

[e] Model 3 further adjusts for exercise, health care coverage, BMI, packyears of smoking, and CESD; median age estimations with exercise and health care coverage fixed at the sample majority (none and presence of healthcare coverage, respectively) and sample mean for BMI (29.9), packyears of smoking (9.4), and CESD (1.4), in addition to specifications listed for Model 2.

[f] Models additionally include robust variance estimators to account for clustering by census tract and fully adjusted models (Model 3) additionally control for neighborhood rurality (estimations fixed at the sample majority—urban residence, in addition to specifications listed for Overall Models 2 and 3).

eTable 4. Estimated Median Age of Hypertension Onset and Differences in Black and White Women Across Levels of Neighborhood Disinvestment by Moving Status, the Reasons for Geographic and Racial Differences in Stroke Study[a]

	**Movers** N=3,708		**Non-movers** N=11,601	
	Age of onset, Median (95% CI)	Difference (95% CI)	Age of onset, Median (95% CI)	Difference (95% CI)
**Neighborhood Disinvestment**				
Low				
Black	58.0 (55.3, 60.7)	-8.5 (-10.7, -6.4)	55.4 (53.9, 57.0)	-6.0 (-7.1, -4.8)
White	66.5 (64.0, 69.0)	Ref	61.4 (59.8, 63.0)	Ref
Moderate				
Black	53.1 (50.2, 55.9)	-7.2 (-9.3, -5.2)	57.4 (55.9, 59.0)	-7.0 (-8.2, -5.9)
White	60.3 (57.2, 63.5)	Ref	64.5 (62.7, 66.3)	Ref
High				
Black	52.9 (49.7, 56.1)	-6.3 (-8.5, -4.0)	55.5 (53.7, 57.2)	-6.6 (-7.9, -5.3)
White	59.2 (55.4, 62.9)	Ref	62.1 (59.9, 64.3)	Ref

[a] Estimates are from accelerated failure time models with age as the time scale and robust variance estimators to account for clustering adjusted for income, education, employment, exercise, health care coverage, BMI, packyears of smoking, CESD, and neighborhood rurality. Median age estimations were determined with income, education, and employment, exercise, health care coverage, and rurality fixed at the sample majority ($35,000-74,999, college graduate or more, retired, no exercise, presence of healthcare coverage, and urban residence, respectively) and sample mean for BMI (29.9), packyears of smoking (9.4), and CESD (1.4). 95% confidence intervals were calculated using the Delta Method.
