# Supplementary material for: Neighborhood Disinvestment and Racial Disparities in Early Hypertension Onset Among Women
Source: JAMA Netw Open. 2026 Jun 23;9(6):e2619845. doi: 10.1001/jamanetworkopen.2026.19845 (PMC13291848; doi:10.1001/jamanetworkopen.2026.19845)
Supplement: Supplement 2. — Data Sharing Statement [file jamanetwopen-e2619845-s002.pdf]

Data Sharing Statement

Hailu. Neighborhood Disinvestment and Racial Disparities in Early Hypertension Onset Among Women. *JAMA Netw Open*. Published June 23, 2026.
doi:10.1001/jamanetworkopen.2026.19845

Data
Data available: No

Additional Information
Explanation for why data not available: Investigators trained in human subjects research may direct inquiries about access to the Reasons for Geographic and Racial Differences in Stroke Study at https://www.uab.edu/soph/regardsstudy/
